# Integrative Multi-Omics Analysis for the Determination of Non-Muscle Invasive vs. Muscle Invasive Bladder Cancer: A Pilot Study

**DOI:** 10.3390/curroncol29080430

**Published:** 2022-07-31

**Authors:** Evan Yi-Wen Yu, Hao Zhang, Yuanqing Fu, Ya-Ting Chen, Qiu-Yi Tang, Yu-Xiang Liu, Yan-Xi Zhang, Shi-Zhi Wang, Anke Wesselius, Wen-Chao Li, Maurice P. Zeegers, Bin Xu

**Affiliations:** 1 Key Laboratory of Environmental Medicine and Engineering of Ministry of Education, Department of Epidemiology & Biostatistics, School of Public Health, Southeast University, Nanjing 210009, China; ytcchen1997@hotmail.com (Y.-T.C.); npliuyi1@gmail.com (Y.-X.L.); zhangzhang_1020@163.com (Y.-X.Z.); 2 Department of Epidemiology, CAPHRI Care and Public Health Research Institute, Maastricht University, 6229 ER Maastricht, The Netherlands; anke.wesselius@maastrichtuniversity.nl (A.W.); m.zeegers@maastrichtuniversity.nl (M.P.Z.); 3 State Key Laboratory of Bioelectronics, National Demonstration Center for Experimental Biomedical Engineering Education, Southeast University, Nanjing 210096, China; 4 Nanjing EVLiXiR Biotechnology Co., Ltd., Nanjing 210032, China; 5 Key Laboratory of Growth Regulation and Translational Research of Zhejiang Province, School of Life Sciences, Westlake University, Hangzhou 310024, China; fuyuanqing@westlake.edu.cn; 6 Westlake Intelligent Biomarker Discovery Laboratory, Westlake Laboratory of Life Sciences and Biomedicine, Hangzhou 310024, China; 7 Institute of Basic Medical Sciences, Westlake Institute for Advanced Study, Hangzhou 310024, China; 8 Medical School of Southeast University, Nanjing 210009, China; hsyxtqy@gmail.com; 9 Key Laboratory of Environmental Medicine Engineering, School of Public Health, Southeast University, Ministry of Education, Nanjing 210009, China; shizhiwang2009@seu.edu.cn; 10 School of Nutrition and Translational Research in Metabolism, Maastricht University, 6229 ER Maastricht, The Netherlands; 11 Department of Urology, Affiliated Zhongda Hospital of Southeast University, Nanjing 210009, China; liwenchao_lee@hotmail.com (W.-C.L.); njxb1982@126.com (B.X.)

**Keywords:** bladder cancer, multi-omics, progression of disease, molecular epidemiology

## Abstract

Objectives: The molecular landscape of non-muscle-invasive (NMIBC) and muscle-invasive (MIBC) bladder cancer based on molecular characteristics is essential but poorly understood. In this pilot study we aimed to identify a multi-omics signature that can distinguish MIBC from NMIBC. Such a signature can assist in finding potential mechanistic biomarkers and druggable targets. Methods: Patients diagnosed with NMIBC (*n* = 15) and MIBC (*n* = 11) were recruited at a tertiary-care hospital in Nanjing from 1 April 2021, and 31 July 2021. Blood, urine and stool samples per participant were collected, in which the serum metabolome, urine metabolome, gut microbiome, and serum extracellular vesicles (EV) proteome were quantified. The differences of the global profiles and individual omics measure between NMIBC vs. MIBC were assessed by permutational multivariate analysis and the Mann–Whitney test, respectively. Logistic regression analysis was used to assess the association of each identified analyte with NMIBC vs. MIBC, and the Spearman correlation was used to investigate the correlations between identified analytes, where both were adjusted for age, sex and smoking status. Results: Among 3168 multi-omics measures that passed the quality control, 159 were identified to be differentiated in NMIBC vs. MIBC. Of these, 46 analytes were associated with bladder cancer progression. In addition, the global profiles showed significantly different urine metabolome (*p* = 0.029), gut microbiome (*p* = 0.036), and serum EV (extracellular vesicles) proteome (*p* = 0.039) but not serum metabolome (*p* = 0.059). We also observed 17 (35%) analytes that had been developed as drug targets. Multiple interactions were obtained between the identified analytes, whereas for the majority (61%), the number of interactions was at 11–20. Moreover, unconjugated bilirubin (*p* = 0.009) and white blood cell count (*p* = 0.006) were also shown to be different in NMIBC and MIBC, and associated with 11 identified omics analytes. Conclusions: The pilot study has shown promising to monitor the progression of bladder cancer by integrating multi-omics data and deserves further investigations.

## 1. Introduction

Bladder cancer (BC) is the most common urinary tract malignancy with approximately 550,000 new cases and 200,000 deaths worldwide yearly [1,2]. Urothelial cell carcinoma is the predominant histological type, which is often classified as non-muscle-invasive bladder cancer (NMIBC), or muscle-invasive bladder cancer (MIBC) depending on whether the tumour has invaded into the muscularis propria. Although only about 25% of newly diagnosed patients present with muscle invasive disease, MIBC has accounted for the majority of bladder cancer mortality [3]. Clinically, the 5-year survival rate of MIBC is <15% despite the applications of advanced therapies over the past decades [4], and requires frequent physical examinations, making bladder cancer the most expensive cancer to treat [5]. An in-depth study of the molecular characteristics of different subtypes (i.e., NMIBC vs. MIBC) is therefore essential for the development of biomarkers that can target tumour progression.

In recent years, many studies have characterised the molecular characteristics at different omics levels and have resulted in the identification of promising novel biomarkers and therapeutic targets, which could improve outcomes for patients with this disease [6]. Nevertheless, the previous studies that investigated the molecular profiles NMIBC vs. MIBC were only based on single-omics data [7,8,9,10,11], and therefore the complexity of bladder cancer were not fully captured. In addition, the measurement of omics data differs in sample types [12], which causes the challenge in detecting the markers with low concentrations. Thus, we need better biomarkers, especially those up- or down-regulated consistently in NMIBC or MIBC, to increase the opportunities in identifying the early stages of bladder cancer progression. Furthermore, although recent advances in high-throughput omics measurement and bioinformatics analysis have provided the platforms and opportunities for the discovery of new bladder cancer biomarkers and druggable targets [13], there is currently a lack of comprehensive molecular profiling studies focusing on the downstream molecules, i.e., metabolites and proteins. In addition to metabolomic or proteomic markers, extracellular vesicles (EV) and gut microbiota are also useful and novel biomarker candidates due to their essential role in various pathways [14,15]; however, the studies investigating the EV and gut microbiota in relation to bladder cancer are largely scarce.

Here, we initiated a population-based study, which aimed to perform a comprehensive analysis of multi-omics including the serum metabolome, urine metabolome, stool metagenome, and serum EV proteome, with the goal of identifying biomarkers and their interactions to distinguish the NMIBC vs. MIBC patients. These results can provide preliminarily evidence and support the further study for the molecular subtyping of bladder cancer from the perspective of multi-omics.

## 2. Materials and Methods

**Figure 1:**
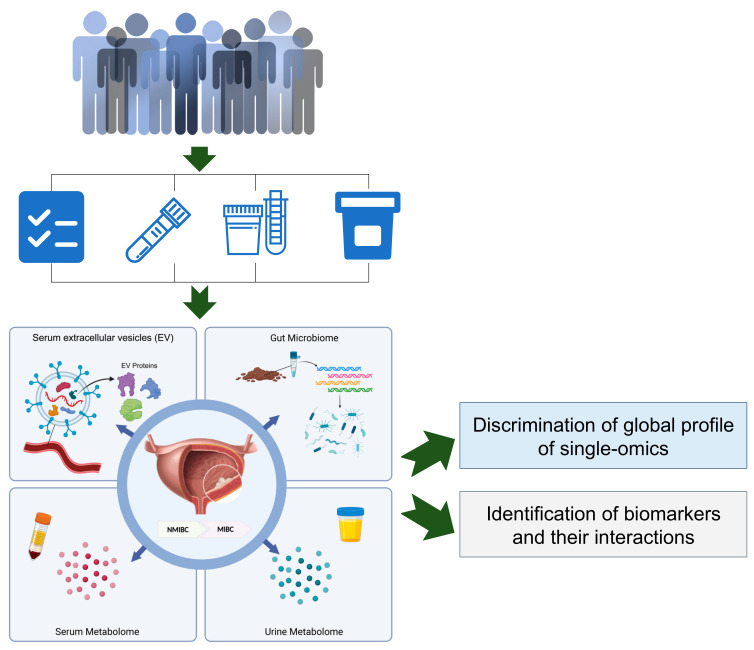
Design and work-flow of the current study. The NMIBC and MIBC patients were recruited with specimen collection for blood, urine and stool. Abbreviations: NMIBC, non-muscle-invasive bladder cancer; MIBC, muscle-invasive bladder cancer.

To obtain a brief landscape of multi-omics for patients with NMIBC and MIBC, we designed a pilot study with measurements in laboratory tests and multi-omics (i.e., the serum metabolome, urine metabolome, serum EV proteome and gut microbiome), as presented in Figure 1.

### 2.1. Participants Recruitment and Demographic Characteristics Assessment

All participants were informed about the study’s purpose during the admission interview, and voluntarily informed consent forms prior to enrolling in the study. All procedures in this study were compliant with the Declaration of Helsinki, and the study’s protocol was approved by the Ethics Committee of the Zhongda Hospital of Southeast University, Nanjing, China.

Bladder cancer patients were recruited for this study at a tertiary-care hospital in Nanjing from between 1 April 2021, and 31 July 2021. All the BC patients were local residents, and of Han ancestry. The medical records of each patient were reviewed by trained doctors or nurses, and clinicopathological characteristics of bladder cancers at the diagnosis were prospectively gathered on dedicated case report forms. None of the patients were undergoing other medical interventions. The diagnosis and progression of bladder cancer was confirmed with histology, in which the tumours were re-staged according to the 2009 American Joint Committee on TNM (Tumour, Node and Metastasis) classification of bladder tumours and graded according to the 2004 World Health Organization grading scheme [16]. In addition, all the specimens (i.e., blood, urine, and stool) were collected before the patients went to surgery or chemotherapy. In total, 26 bladder cancer patients were included into the current study: 15 were NMIBC patients and 11 were MIBC patients.

### 2.2. Sample Collection and Quantification

Whole blood samples were collected after an overnight fast, stored for about 30 min in room temperature before sending the sample to the clinical chemistry laboratory using a biosafety transport box. The laboratory obtained the sample and centrifuged them at 1500× *g* for 10 min. Then, we collected the serum in new centrifuge tubes and immediately stored them at −80 °C. Meanwhile, the urine samples were thawed in a thermostatically controlled water bath at 37 °C and immediately centrifuged at 3900× *g* for 10 min. The fresh stool samples were collected into sterilised and portable plastic containers with waxed tissue paper (Epitope Diagnostics, San Diego, CA, USA), and then were transferred to a −80 °C facility within 4 h after collection.

### 2.3. Metabolome Measurement for Serum and Urine Sample

The blood sample was taken out from the −80 °C refrigerator and thawed on ice, then vortexed for 10 s. We mixed 50 μL of sample and 300 μL of 20% acetonitrile methanol internal standard extractant, vortexed the mixture for 3 min and centrifuged (8000× *g*, 4 °C) for 10 min. Then, we transferred 200 μL of the supernatant and stored it at −20 °C for 30 min. Finally, we centrifuged (8000× *g*, 4 °C) the sample for 3 min and took the supernatant for analysis. For the urine samples, they were taken out from the −80 °C refrigerator and thawed on ice, then vortexed for 10 s. Then, we mixed 200 μL of sample and 200 μL of 20% acetonitrile methanol internal standard extractant, vortexed the mixture for 3 min and centrifuged (8000× *g*, 4 °C) for 10 min. Then, 350 μL of the supernatant was transferred and dried. We reconstituted the dry residue with 150 μL of 70% methanol water, vortexed for 3 min, and sonicated it for 10 min in ice water bath. Finally, it was centrifuged (8000× *g*, 4 °C) for 3 min and the supernatant was taken for analysis.

The blood sample was taken out from the −80 °C refrigerator and thawed on ice until the sample is free of ice. The sample was vortexed for 10 s. Then 50 μL of sample was mixed with 300 μL of 20% acetonitrile methanol internal standard extractant, which was vortexed for 3 min, and centrifuged at 12,000 r/min for 10 min at 4 °C. After centrifugation, 200 μL of the supernatant was transferred into a centrifuge tube and stored for 30 min at −20 °C in a refrigerator. Finally, the sample was centrifuged (8000× *g*, 4 °C) for 3 min and then 180 μL of supernatant was taken for analysis. For the urine sample, similarly, the sample was taken out from the −80 °C refrigerator and thawed on ice until the sample was free of ice. The sample was vortexed for 10 s. Then, 200 μL of sample was mixed with 200 μL of 20% acetonitrile methanol internal standard extractant, which was vortexed for 3 min, and centrifuged at 12,000 r/min for 10 min at 4 °C. Then, 350 μL of the supernatant was transferred and dried. The dry residue was reconstituted with 150 μL of 70% methanol water, vortexed for 3 min, and sonicated for 10 min in an ice water bath. We reconstituted the dry residue with 150 μL of 70% methanol water, vortexed it for 3 min, and sonicated it for 10 min in an ice water bath. Finally, the sample was centrifuged (8000× *g*, 4 °C) for 3 min and then 120 μL of supernatant was taken for analysis.

The metabolome was measured according to the procedures and conditions described as follows; (1) T3 UPLC Conditions: the sample extracts were analysed using an LC-ESI-MS/MS system (UPLC, ExionLC AD 1.7, Framinghan, MA, USA https://sciex.com.cn/, accessed on 15 October 2021; MS, QTRAP^®^ System, Framinghan, MA, USA, https://sciex.com/, accessed on 15 October 2021). The analytical conditions were as follows, UPLC: column, Waters ACQUITY UPLC HSS T3 C18 (1.8 µm, 2.1 mm × 100 mm); column temperature, 40 °C; flow rate, 0.4 mL/min; injection volume, 2 μL; solvent system, water (0.1% formic acid): acetonitrile (0.1% formic acid); gradient program, 95:5 V/V at 0 min, 10:90 V/V at 11.0 min, 10:90 V/V at 12.0 min, 95:5 V/V at 12.1 min, 95:5 V/V at 14.0 min; (2) Amide UPLC Conditions: The sample extracts were analysed using an LC-ESI-MS/MS system (UPLC, ExionLC AD, 1.7, Framinghan, MA, USA, https://sciex.com.cn/, accessed on 15 November 2021; MS, QTRAP^®^ System, Framinghan, MA, USA, https://sciex.com/, accessed on 15 November 2021). The analytical conditions were as follows, UPLC: column, Waters ACQUITY UPLC BEH Amide 1.7 µm, 2.1 mm × 100 mm; column temperature, 40 °C; flow rate, 0.4 mL/min; injection volume, 2 μL; solvent system, water (25 mM Ammonium formate/0.4% Ammonia): acetonitrile; gradient program, 10:90 V/V at 0 min, 40:60 V/V at 9.0 min, 60:40 V/V at 10.0 min, 60:40 V/V at 11.0 min, 10:90 V/V at 11.1 min, 10:90 V/V at 15.0 min; (3) ESI-QTRAP-MS/MS: T3 and Hilic have the same mass spectrometry parameters. LIT and triple quadrupole (QQQ) scans were acquired on a triple quadrupole-linear ion trap mass spectrometer (QTRAP), QTRAP^®^ LC-MS/MS System, equipped with an ESI Turbo Ion-Spray interface, operating in positive and negative ion mode, and controlled by Analyst 1.6.3 software (Sciex). The ESI source operation parameters were as follows: source temperature 500 °C; ion spray voltage (IS) 5500 V (positive), −4500 V (negative); ion source gas I (GSI), gas II (GSII), and curtain gas (CUR) were set at 55, 60, and 25.0 psi, respectively; the collision gas (CAD) was high. Instrument tuning and mass calibration were performed with 10 and 100 μmol/L polypropylene glycol solutions in QQQ and LIT modes, respectively. A specific set of MRM transitions were monitored for each period according to the metabolites eluted within this period.

### 2.4. Extracellular Vesicles (EV) Proteome Measurement for Serum Sample

For EV proteome analysis, the serum samples obtained from patients with NMIBC and MIBC individuals were pooled together. These two groups of pooled serum samples were used for EV isolation and quantified by using a previously published protocol [17]; (1) preparation of serum: a serum sample was diluted using phosphate-buffered solution (PBS), and centrifuged at 500× *g* for 10 min at 4 °C to remove cells and dead cells; the supernatant was collected and centrifuged at 2000× *g* for 10 min at 4 °C to remove cell debris; the supernatant was collected and centrifuged at 10,000× g for 30 min at 4 °C to remove large vesicles; the supernatant was collected and centrifuged continuously at 100,000× g for 90 min at 4 °C to remove the supernatant; then, the leftover was centrifuged at 100,000× g for 90 min at 4 °C, and the supernatant was removed. EVs were obtained by resuspending the leftover precipitate in PBS and centrifuging again at 100,000× g for 90 min; (2) EV proteolysis: the collected EVs were added into 100 μL PTS lysate and then put in a water bath at 95 °C for 10 min. Then, it was cooled to room temperature and added with 450 μL of 50 mM TEAB buffer. Lys-C enzyme was added according to the sample protein content:enzyme = 100:1 and incubated at 37 °C for 3 h. The trypsinase enzyme was added according to sample protein content:trypsin = 50:1 and incubated at 37 °C for 16 h. An amount of 50 μL of 10% TFA was used to terminate the enzymatic digestion. Five times the volume of ethyl acetate was added and vortexed for 2 min, centrifuged at 12,000× *g* for 3 min, then we removed the upper layer of ethyl acetate, and repeated the process once. The samples were lyophilised, then the lyophilised samples were desalted and desalted pending mass spectrometric detection; (3) LC–MS/MS and data analysis: the desalted samples were re-solubilised using 0.1% formic acid, and appropriate amounts of peptides were taken from each case for chromatographic separation using a nanolitre flow rate Easy-nLC 1200 chromatography system (Thermo Scientific, Waltham, MA, USA). Buffer: Solution A was 0.1% formic acid in water and Solution B was 80% ACN/0.1% formic acid. The chromatographic column was equilibrated with 100% of liquid A. The sample was fed and passed through the chromatographic analytical column for gradient separation at a flow rate of 300 nL/min. The liquid phase separation gradients were as follows; 0–3 min, linear gradient of B liquid from 2 to 8%; 3–81 min, linear gradient of B liquid from 8 to 40%; 81–83 min, linear gradient of B liquid from 40 to 95%; 83–90 min, B liquid maintained at 95%. The peptides were separated and analysed by DIA (data independent acquisition) mass spectrometry using a Q-Exactive HF-X mass spectrometer (Thermo Scientific). Analysis time was 90 min, detection mode: positive ion, parent ion scan range: 390–1210 *m*/*z*, primary mass resolution: 60,000, AGC target: 1e6, primary maximum IT: 60 ms. DIA method acquisition: 75 secondary mass spectra of the highest intensity parent ions were triggered after each full scan (full scan) MS2 scan, secondary MS resolution: 15,000, AGC target: 1e6, Maximum IT: 20 ms, Isolation window: 8 *m*/*z*, Normalized collision energy: 27. The mass spectrometry data were analysed using the directDIA analysis function of Spectronaut™ software 16 (Schlieren, Switzerland), using the human source database downloaded from the Uniprot website (https://www.uniprot.org, accessed on 22 March 2022) as the database for directDIA analysis, and the search library parameters were the default parameters of the BGS Factory Setting for the directDIA analysis process.

### 2.5. Metagenome Measurement from Stool Sample

Stool DNA extractions were carried out by a standardised CTAB procedure. DNA concentration was measured using the Qubit dsDNA Assay Kit in Qubit 2.0 Fluorometer (Life Technologies, Carlsbad, CA, USA). For DNA library preparation, a total amount of 1 μg DNA per sample was used as input material. In addition, the NEBNext Ultra DNA Library Prep Kit (NEB, Ipswich, MA, USA) was used following the manufacturer’s recommendations and index codes were added to attribute sequences to each sample. The DNA samples were fragmented by sonication to a size of approximately 350 bp. Then, the DNA fragments were end-polished, A-tailed, and ligated with the full-length adaptor for Illumina sequencing with further PCR amplification. After that, PCR products were purified (AMPure XP system) and libraries were analysed for size distribution by an Agilent2100 Bioanalyzer and quantified using real-time PCR. The clustering of the index-coded samples was performed on a cBot Cluster Generation System according to the manufacturer’s instructions. After cluster generation, the library preparations were sequenced on an Illumina HiSeq platform and 150 bp paired-end reads were generated. Finally, we obtained on average 42.4 million paired-end raw reads for each sample.

Next, raw sequencing reads were first quality-controlled with PRINSEQ (v0.20.4)8: (1) to trim the reads by quality score from the 5′ end and 3′ end with a quality threshold of 20; (2) remove read pairs when either read was <60 bp, contained “N” bases or had a quality score mean below 30; and (3) deduplicate the reads. Reads that could be aligned to the human genome (*H. sapiens*, UCSC hg19) were removed (aligned with Bowtie2 v2.2.5 using –reorder–no-contain–dovetail).

Functional profiling was performed with HUMAnN2 v2.8.19 (Boston, MA, USA), which maps sample reads against the sample-specific reference database to quantify gene presence and abundance in a species-stratified manner, with unmapped reads further used in a translated search against Uniref90 to include taxonomically unclassified but functionally distinct gene family abundances. We extracted the Uniref90 gene families of gut bacteria for downstream analyses. The Uniref90 gene families were then converted into KEGG Orthologs (KOs). The abundances of KOs were normalised into relative abundance for each sample. In addition, the KOs presented in less than 10% of the 1009 samples were excluded from the downstream analysis.

### 2.6. Data Quality Control

The raw data were gained for the serum metabolome (*n* = 782), urine metabolome (*n* = 936), serum EV proteome (*n* = 1491), gut microbiome (*n* = 589), then the quality of omics data was ensured at multiple steps separately. Each omics matrix contained missing values. Missing values can be due to the low abundance in certain samples or technical issues. Firstly, to remove the single-omics data with poor quality, we excluded the data with identifications missing in over the 50% of the participants. Subsequently, we imputed the missing values with 1/2 lowest values measured in each variable of the omics matrix separately. Totally, 3168 measures (including 782 serum metabolites, 936 urine metabolites, 1323 serum EV proteins and 127 gut microbes) remained eligible to be included in the further study.

### 2.7. Statistical and Bioinformatic Analysis

All statistical analyses were performed using Stata 15.0 (Stata Corp., College Station, TX, USA) or R (version 4.0.5). Descriptive statistics are presented as mean (±SD (standard deviation)) or median (interquartile range) for continuous variables, and frequency (percentage, %) for categorial variables (Table 1). Principal coordinates (PCoA) analysis was used, with a permutational multivariate analysis of variance, to perform the difference of global profiles. Statistical difference between the two groups (NMIBC vs. MIBC) was assessed with the Mann–Whitney test. We used logistic regression analysis to assess the association between each identified analyte and bladder cancer progression, with adjustments of age (years, continuous), sex (male and female) and smoking status (never, former, and current smoker). The KEGG (https://www.genome.jp/kegg/, accessed on 16 April 2022) and DrugBank (https://go.drugbank.com, accessed on 16 April 2022) databases were used to annotate the mechanistic pathways and druggable targets of identified analytes. Then, we used the Spearman correlation to investigate the correlations between the secondary pairs of identified analytes, adjusted for age, sex and smoking status. A *p* value < 0.05 was considered statistically significant.

## 3. Results

### 3.1. General Characteristics and Global Profile

**Figure 2:**
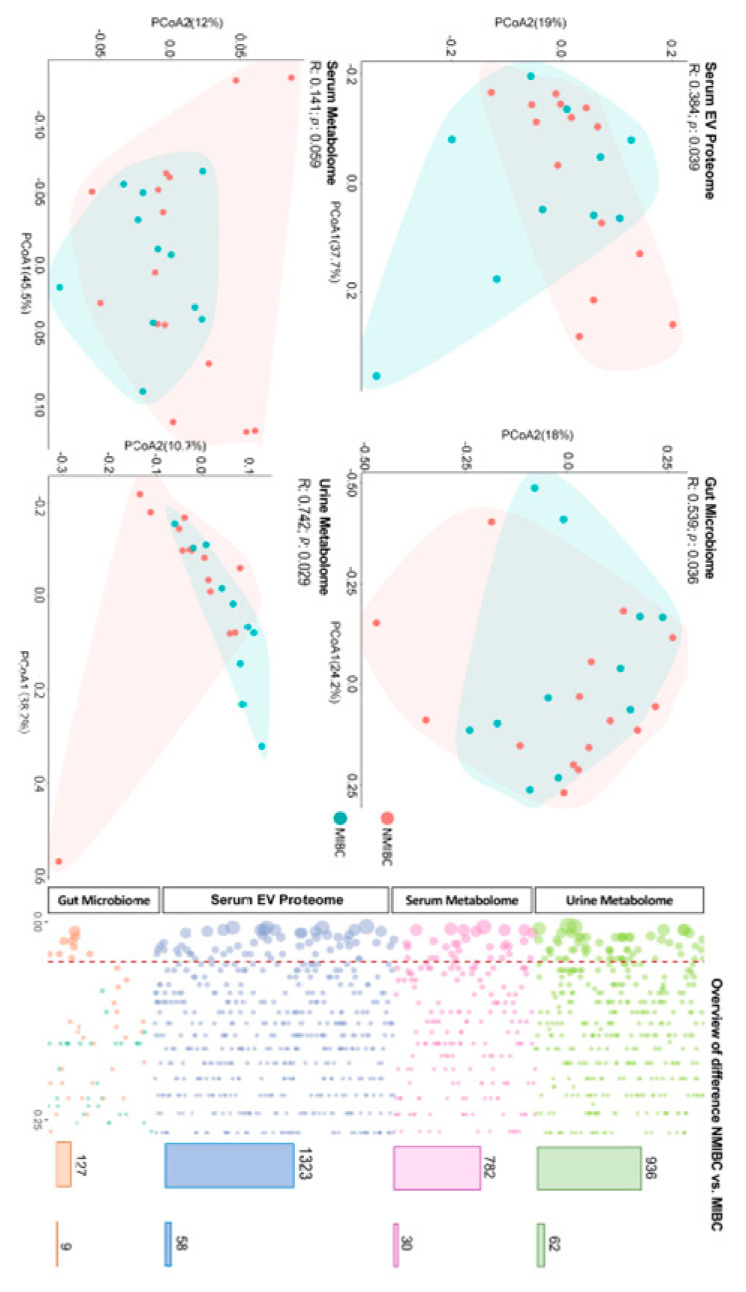
Global profile between NMIBC and MIBC based on multi-omics. To distinguish the molecular characteristics between NMIBC and MIBC, we measured the serum metabolome, urine metabolome, gut microbiome and serum EV proteome. We also profiled the clinical information of each participant. PCoA analysis was used, with a permutational multivariate analysis of variance, to perform the difference of global profiles. Statistical difference between two groups (NMIBC vs. MIBC) was assessed with the Mann–Whitney test. Abbreviations: NMIBC, non-muscle-invasive bladder cancer; MIBC, muscle-invasive bladder cancer; PCoA, Principal coordinates.

Overall, the NMIBC and MIBC patients showed a difference in unconjugated bilirubin (*p* = 0.009) and white blood cell count (*p* = 0.006), while no difference was found for age, sex, smoking status, and other laboratory tests. After quality control, we maintained 3168 multi-omics features, including 1323 serum exosomes, 782 serum metabolites, 936 urine metabolites and 127 gut microbiota. Of those, the global profile showed significant different patterns as revealed by the PCoA analysis on the serum EV proteome (*p* = 0.039), gut microbiome (*p* = 0.036), and urine metabolome (*p* = 0.029) but not in the serum metabolome (*p* = 0.059) (Figure 2).

### 3.2. Difference of NMIBC vs. MIBC According to Individual Omics Measures

**Figure 3:**
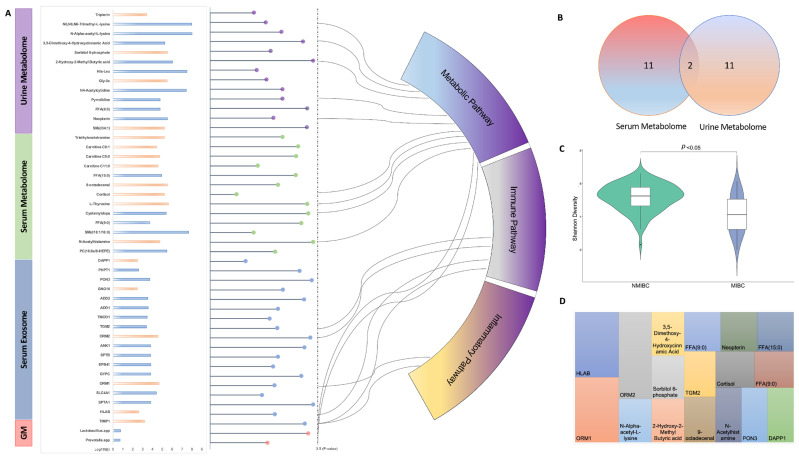
Identification of analytes of multi-omics and functional annotation. (**A**) Identified multi-omics analytes with annotated mechanistic pathways; (**B**) Venn plot of identified metabolites in NMIBC and MIBC; (**C**) Comparison of Shannon diversity between NMIBC and MIBC; (**D**) Established druggable targets from the identified analytes. Abbreviations: EV, extracellular vesicles; GM, gut microbiome; His-leu, Histidine-Leucine; Gly-Ile, Glycyl-Isoleucine; FFA (9:0), Nonanoic acids; SM(d18:1/18:0), Sphingomyelin; FFA (15:0), Pentadecylic acid; DAPP1, Dual adapter for phosphotyrosine and 3-phosphotyrosine and 3-phosphoinositide; PHPT1, 14 kDa phosphohistidine phosphatase; PON3, Serum paraoxonase/lactonase 3; GNG10, Guanine nucleotide-binding protein G(I)/G(S)/G(O) subunit gamma-10; ADD2, Beta-adducin; ADD1, Alpha-adducin; TGM2, Protein-glutamine gamma-glutamyltransferase 2; ORM2, Alpha-1-acid glycoprotein 2; ANK1, Ankyrin-1 OS = *Homo sapiens*; SPTB, Spectrin beta chain non-erythrocytic 1; EPB41, Protein 4.1 OS = *Homo sapiens*; GYPC, Glycophorin-C OS = *Homo sapiens*; ORM1, Alpha-1-acid glycoprotein 1; SLC4A1, Band 3 anion transport protein; SPTA1, Spectrin alpha chain erythrocytic 1; HLAB, HLA class I histocompatibility antigen B alpha chain; Metalloproteinase inhibitor 1.

To determine the essential features that were related to the progression of bladder cancer, we identified 46 analytes with different abundance in NMIBC vs. MIBC, including 18 serum EV proteins, 13 serum metabolites, 13 urine metabolites and 2 gut microbes, as presented in Figure 3A. Of these, 18 analytes showed a higher abundance in MIBC when compared to NMIBC, while 28 analytes had a lower abundance in MIBC when compared to NMIBC. Through a mechanistic mapping, we found 17 (35%) analytes were enriched in well-reported pathways that were categorised to metabolic [11], immune [4] and inflammatory [18] responses. Amongst these, the metabolites derived from serum and urine were largely non-overlapped, while only two metabolites, i.e., nonanoic acid (C9:0) and Sphingomyelin (SM (d18:1/18:0)), showed a significant difference in both serum and urine (Figure 3B). In addition, though the Shannon diversity index was higher in NMIBC when compared to MIBC (*p* < 0.001), we only found two gut microbes at the genus level (i.e., *Lactobacillus* spp. and *Prevotella* spp.) which were lower in MIBC (Figure 3C). Moreover, we found 17 (35%) analytes that had been developed as drug targets based on the DrugBank database (v5.1.8), while others (75%) remained unavailable and deserved further development (Figure 3D).

### 3.3. A Multi-Omics Interaction Map with Responses to Unconjugated Bilirubin and White Blood Cell Count

**Figure 4:**
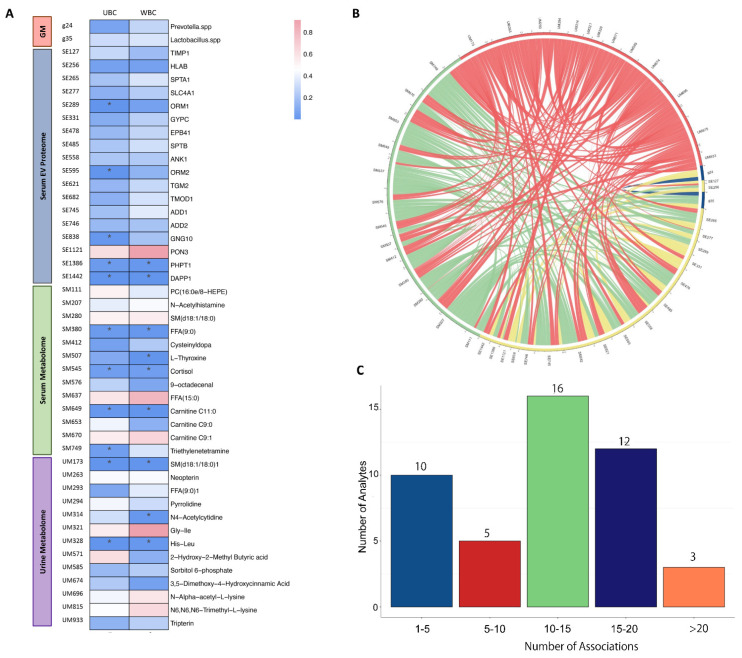
Interactive analysis of identified multi-omics biomarkers. (**A**) Correlation of identified analytes with unconjugated bilirubin and white blood cell count; (**B**) Correlation between each pair of identified analytes; (**C**) The number of correlations of each analyte to others. Abbreviations: UBC, unconjugated bilirubin; WBC, white blood cell count; His-leu, Histidine-Leucine; Gly-Ile, Glycyl-Isoleucine; FFA (9:0), Nonanoic acids; SM (d18:1/18:0), Sphingomyelin; FFA (15:0), Pentadecylic acid; DAPP1, Dual adapter for phosphotyrosine and 3-phosphotyrosine and 3-phosphoinositide; PHPT1, 14 kDa phosphohistidine phosphatase; PON3, Serum paraoxonase/lactonase 3; GNG10, Guanine nucleotide-binding protein G(I)/G(S)/G(O) subunit gamma-10; ADD2, Beta-adducin; ADD1, Alpha-adducin; TGM2, Protein-glutamine gamma-glutamyltransferase 2; ORM2, Alpha-1-acid glycoprotein 2; ANK1, Ankyrin-1 OS = *Homo sapiens*; SPTB, Spectrin beta chain non-erythrocytic 1; EPB41, Protein 4.1 OS = *Homo sapiens*; GYPC, Glycophorin-C OS = *Homo sapiens*; ORM1, Alpha-1-acid glycoprotein 1; SLC4A1, Band 3 anion transport protein; SPTA1, Spectrin alpha chain erythrocytic 1; HLAB, HLA class I histocompatibility antigen B alpha chain; Metalloproteinase inhibitor 1. * *p* < 0.05.

According to the difference of unconjugated bilirubin and white blood cell counts that was shown to be significant in the comparison between NMIBC and MIBC, this indicated a potential biomarker for the progression of bladder cancer. We, therefore, performed a correlation analysis of each significant omics analytes to unconjugated bilirubin and white blood cell counts using the Spearman rank method, where four (ORM1 (Alpha-1-acid glycoprotein 1), ORM2 (Alpha-1-acid glycoprotein 2), GNG10 (Guanine nucleotide-binding protein G(I)/G(S)/G(O) subunit gamma-10) and Triethylenetetramine) and two (i.e., L-Thyroxine and N4-Acetylcytidine) analytes were uniquely associated with unconjugated bilirubin and white blood cell counts, respectively, and seven analytes (i.e., PHPT1 (phosphohistidine phosphatase 1), DAPP1 (Dual Adaptor of Phosphotyrosine and 3-Phosphoinositides 1), Nonanoic acids (C9:0), Cortisol, Creatine C11:0, Sphingomyelin (SM (d18:1/18:0)) and Histidine–Leucine) were associated with both (Figure 4A). Where we identified the significant single-omics analytes associated with NMIBC vs. MIBC, the associations of each analyte with others are yet unappreciated. Using Spearman rank correlations, we generated the interaction map between each pair of analytes; the results revealed multiple interactions for a single-omics analyte with others, in which the majority (61%) had a number of interactions ranging from 11 to 20 (Figure 4B,C).

## 4. Discussion

In the current pilot study, we report the single- and multi-omics signatures in response to the progression of bladder cancer (i.e., NMIBC vs. MIBC). We observed distinct global profiles of single-omics, with an identification of 46 analytes showing a significantly differentiated abundance for NMIBC vs. MIBC. While this study was not designed to identify causal links between molecules and bladder cancer prognosis, the results provide mechanistic and therapeutic benchmarks of multi-omics for the management and prevention of progression of bladder cancer, which need to be verified in future studies.

Though few studies have investigated the relationships between gut and bladder, recent studies have indicated a “gut–bladder axis” linking those two distal organs [19,20], where gut microbiota may play a pivotal role. With the emergence of accumulative evidence, gut microbiome has been reported to be associated with cancer occurrence and development; however, the spectrum of direct and indirect interactions between the gut microbiota and the bladder cancer remains largely unknown. Two studies conducted in Japan demonstrated a promising strategy for preventing the recurrence of bladder cancer using *Lactobacillus* spp. as an intervention, which partially supports the observation in our study that the abundance of *Lactobacillus* spp. was lower in MIBC when compared to NMIBC. Experimental studies have revealed that *Lactobacillus* spp. can induce apoptosis in cancer cells via the activation of pro-caspases and pro-apoptotic Bax and the inactivation of the anti-apoptotic Bcl-2 proteins [21,22], which may reduce chemotherapy toxicity and thereby might inhibit the progression of bladder cancer. In addition, we also identified *Prevotella* spp. as a distinct marker for bladder cancer progression in the current study, with a lower abundance in MIBC. Though there is no study yet to provide evidence of gut *Prevotella* spp. related to bladder cancer progression, a study reported a decreased abundance in bladder cancer patients when compared to healthy controls based on a case–control design [23]. Emerging studies in humans have linked the increased abundance of *Prevotella* spp. in relation to reduction of inflammation, and thereby may be clinically important pathobionts that can participate in human disease by promoting chronic inflammation [24]. To our knowledge, inflammation, along with separate arms of the host immune system, not only plays an important role in the development and progression of many different diseases, particularly cancers, but also serves as an important indicator of the prognosis in patients [25]. Further studies are warranted to verify the function and mechanism of *Prevotella* spp. in bladder cancer development.

As an overture of analysing the EV proteome at the circulating level, our study identified 18 serum EV proteins differentiated in NMIBC and MIBC, which were shown to be explosive as novel biomarkers for bladder cancer progression. In recent years, several studies have showed that exosomes, containing proteins, nucleic acids, carbohydrates, and lipids, could provide new non-invasive diagnostic and prognostic biomarkers in patients affected by cancers, including bladder cancer, and the lipid bilayer membrane structure has made exosomes as promising delivery vehicles for therapeutic applications [26]. Of those EV proteins identified above, we found that eight EV proteins have been reported to have an association with certain cancer types, while the remaining 10 ones have yet to be well investigated. Amongst these, SPTA1 (Spectrin alpha chain erythrocytic 1), ORM2 and TMOD1 (Tropomodulin-1) were newly identified and have not been reported to have any association with human health. Hence, their mechanisms in distinguishment and prevention of bladder cancer progression need further investigation.

In line with a previous study [27], our study found a distinguished metabolomic profile based on urine samples, while no difference could be observed for the global profile of the serum metabolome. This finding indicates that urinary metabolomics can be recognised as the preferred approach for biomarker identification of bladder cancer, given that environmental compounds are excreted in urine and thereby come into direct contact with the surface of the bladder [28]. In addition, the metabolites identified in our studies with different abundances in NMIBC and MIBC were largely non-overlapped with only two metabolites co-existing, which suggests that different liquid biopsies may contain different information regarding same omics data and need to be investigated separately. The two metabolites, i.e., Nonanoic acids (C9:0) and Sphingomyelin (SM (d18:1/18:0)), were both negatively associated with the progression of bladder cancer. As one of the odd-chain fatty acids, Nonanoic acid (C9:0) was found to inhibit the expression of HDAC6, which result in an anti-cancer proliferation effect [29]; again, the detailed mechanism needs to be confirmed in bladder cancer. For the Sphingomyelin (SM (d18:1/18:0)), no study has yet fully investigated its effect on cancer, though a study reported it could be related to Alzheimer’s disease [30]. Due to its consistent results showing in both serum and urine, the Sphingomyelin (SM (d18:1/18:0)) may deserve to be further analysed.

Furthermore, this study showed interactions between different kinds of omics across multiple samples, which indicated bladder cancer, as a complex disease, might be affected by systemic factors. However, the network integrating multiple omics still need to be further investigated. Despite this being a pilot study, several limitations arouse our attention; the small sample size and collection provided limited power to assess differential expression in NMIBC and MIBC. With the consideration of sample size, multiple testing was not applied in the current study, which may have resulted in potential false-positive findings. While we identified some indications of single- and multiple omics, our findings warrant further investigations to confirm the biomarkers and their interactions in the context of bladder cancer progression. In addition, while high-throughput techniques offer a high-resolution view of the relevant molecules related to bladder, we cannot rule out the presence of additional, low-abundance molecules that could not be detected from the depth of multi-omics generated.

In summary, the current pilot study was initiated for investigating the progression biomarkers from the perspective of molecular omics data in Asian ancestry, which, based on the preliminarily data, has shown to be promising in monitoring the progression of bladder cancer by integrating multi-omics data. By integrating multiple data with in-depth analysis, the results gained in the current study may contribute to managing the progression, decreasing the health burden, and improving the health-related quality of life of patients with bladder cancer; hence, further research with a larger sample size and data is needed.

## Figures and Tables

**Table 1:** Characteristics of included participants in the bladder cancer pilot study.

Characteristics	MIBC	NMIBC	*p*-Value
	*n* = 11	*n* = 15	
**Male (%)**	9 (80.82%)	14 (93.33%)	0.150
**Age (year)**	71.18 ± 8.64	66.93 ± 10.95	0.298
**Smoking status**			0.644
Never (%)	6 (54.55)	5 (33.33)	
Current (%)	1 (9.09)	2 (13.33)	
Former (%)	4 (36.36)	7 (53.34)	
**Potassium (mmonl/L)**	3.99 (3.55–4.22)	3.74 (3.58–4.01)	0.484
**Sodium (mmonl/L)**	138.70 (136.80–141.70)	140.40 (138.70–143.40)	0.204
**Chlorine (mmonl/L)**	104.05 (102.20–107.50)	104.50 (102.70–109.00)	0.755
**Calcium (mmonl/L)**	2.21 (2.10–2.27)	2.21 (2.15–2.26)	0.979
**Phosphate (mmonl/L)**	1.11 (0.98–1.30)	1.11 (1.06–1.18)	0.678
**CO_2_ (mmonl/L)**	24.80 (22.00–29.00)	27.30 (23.40–29.00)	0.640
**Total Protein (g/L)**	65.05 (63.20–69.80)	66.50 (61.10–68.80)	0.953
**Albumin (g/L)**	38.15 (34.90–40.10)	40.00 (35.20–41.50)	0.598
**Globulin (g/L)**	28.00 (24.80–32.00)	26.85 (22.40–31.60)	0.725
**Ratio of albumin to globulin**	1.27 (1.20–1.38)	1.40 (1.20–1.54)	0.447
**Unconjugated bilirubin (µmol/L)**	5.35 (3.70–8.10)	9.55 (8.10–17.80)	**0.009**
**Conjugated bilirubin (µmol/L)**	1.75 (0.85–3.80)	2.16 (1.55–4.11)	0.598
**Alanine transaminase (U/L)**	15.00 (12.00–38.00)	20.00 (11.00–34.00)	0.619
**Aspartate Transaminase (U/L)**	19.00 (17.00–34.00)	22.00 (20.00–27.00)	0.244
**alkaline phosphatase (U/L)**	80.50 (58.00–96.00)	61.00 (48.00–70.00)	0.107
**Gamma-glutamyl transferase (U/L)**	30.00 (20.00–35.00)	26.50 (17.00–40.00)	0.501
**lactate dehydrogenase (U/L)**	255.50 (195.00–363.00)	383.00 (216.00–410.00)	0.183
**Cholinesterase (U/L)**	6127.00 (5418.00–6989.00)	6203.50 (5609.50–7843.00)	0.394
**oral glucose tolerance test (mmol/L)**	7.27 (5.86–7.80)	5.89 (5.30–7.90)	0.243
**Urea (mmol/L)**	69.00 (59.00–130.00)	78.00 (68.00–90.00)	0.938
**Creatinine (µmol/L)**	69.00 (59.00–130.00)	78.00 (68.00–90.00)	0.622
**Uric acid (µmol/L)**	359.00 (242.00–412.00)	314.00 (225.00–381.00)	0.568
**Red blood cell (/µL)**	556.00 (41.00–5774.00)	154.00 (4.00–4294.00)	0.436
**White blood cell (/µL)**	57.00 (37.00–392.00)	19.00 (4.00–46.00)	**0.006**
**Red blood cell count in urine (10,000/mL)**	55.60 (4.10–577.40)	15.40 (0.40–429.40)	0.436

## Data Availability

The data that support the findings of this study will be available on reasonable request pending approval from the corresponding author, E.Y.-W.Y. The data and code are not publicly available owing to their containing information that could compromise the privacy of research participants.

## Abbreviations

CTAB, cetyltrimethylammonium bromide; DAPP1, Dual Adaptor Of Phosphotyrosine And 3-Phosphoinositides 1; DIA, data independent analysis; EV, extracellular vesicles; GNG10, Guanine nucleotide-binding protein G(I)/G(S)/G(O) subunit gamma-10; HDAC6, Histone deacetylase 6; KEGG, Kyoto Encyclopedia of Genes and Genomes; LC-ESI-MS, Liquid Chromatography-Electrospray Ionization-Mass Spectrometry; MIBC, muscle invasive bladder cancer; MRM, m/z ratio of a peptide and its corresponding product ion *m*/*z* ratio; MS, Mass Spectrometry; NMIBC, non-muscle invasive bladder cancer; ORM1, Alpha-1-acid glycoprotein 1; ORM2, Alpha-1-acid glycoprotein 2; PBS, Phosphate-buffered saline; PHPT1, phosphohistidine phosphatase 1; PCoA, Principal coordinates; PTS, 6-pyruvoyltetrahydropterin synthase; Rpm, Revolutions Per Minute; SPTA1, Spectrin alpha chain erythrocytic 1; TEAB, Triethylammonium bicarbonate; TFA, Trifluoroacetic acid; TMOD1, ropomodulin-1; TNM, Tumour, Node and Metastasis; UPLC, Ultra performance liquid chromatography; UPLC, Ultra performance liquid chromatography.

## References

[1] Ferlay J., Soerjomataram I., Dikshit R., Eser S., Mathers C., Rebelo M., Parkin D.M., Forman D., Bray F. (2015). Cancer incidence and mortality worldwide: Sources, methods and major patterns in GLOBOCAN 2012. Int. J. Cancer.

[2] Siegel R.L., Miller K.D., Fuchs H.E., Jemal A. (2022). Cancer statistics, 2022. CA Cancer J. Clin..

[3] Costello J.C., Theodorescu D. (2014). Decade in review-bladder cancer: International progress: From cytology to genomics. Nat. Rev. Urol..

[4] Abdollah F., Gandaglia G., Thuret R., Schmitges J., Tian Z., Jeldres C., Passoni N.M., Briganti A., Shariat S.F., Perrotte P. (2013). Incidence, survival and mortality rates of stage-specific bladder cancer in United States: A trend analysis. Cancer Epidemiology.

[5] Svatek R.S., Hollenbeck B.K., Holmäng S., Lee R., Kim S.P., Stenzl A., Lotan Y. (2014). The Economics of Bladder Cancer: Costs and Considerations of Caring for This Disease. Eur. Urol..

[6] Mitra A.P., Cote R.J. (2010). Molecular screening for bladder cancer: Progress and potential. Nat. Rev. Urol..

[7] Sjödahl G., Lauss M., Lövgren K., Chebil G., Gudjonsson S., Veerla S., Patschan O., Aine M., Fernö M., Ringnér M. (2012). A Molecular Taxonomy for Urothelial Carcinoma. Clin. Cancer Res..

[8] Cancer Genome Atlas Research Network (2014). Comprehensive molecular characterization of urothelial bladder carcinoma. Nature.

[9] Choi W., Porten S., Kim S., Willis D., Plimack E.R., Hoffman-Censits J., Roth B., Cheng T., Tran M., Lee I.-L. (2014). Identification of Distinct Basal and Luminal Subtypes of Muscle-Invasive Bladder Cancer with Different Sensitivities to Frontline Chemotherapy. Cancer Cell.

[10] Biton A., Bernard-Pierrot I., Lou Y., Krucker C., Chapeaublanc E., Rubio-Pérez C., López-Bigas N., Kamoun A., Neuzillet Y., Gestraud P. (2014). Independent component analysis uncovers the landscape of the bladder tumor transcriptome and reveals insights into luminal and basal subtypes. Cell Rep..

[11] Robertson A.G., Kim J., Al-Ahmadie H., Bellmunt J., Guo G., Cherniack A.D., Hinoue T., Laird P.W., Hoadley K.A., Akbani R. (2017). Comprehensive Molecular Characterization of Muscle-Invasive Bladder Cancer. Cell.

[12] Subramanian I., Verma S., Kumar S., Jere A., Anamika K. (2020). Multi-omics Data Integration, Interpretation, and Its Application. Bioinform. Biol. Insights.

[13] Yuan J., Hegde P.S., Clynes R., Foukas P.G., Harari A., Kleen T.O., Kvistborg P., Maccalli C., Maecker H.T., Page D.B. (2016). Novel technologies and emerging biomarkers for personalized cancer immunotherapy. J. Immunother. Cancer.

[14] Zhang L., Yu D. (2019). Exosomes in cancer development, metastasis, and immunity. Biochim. Biophys. Acta Rev. Cancer.

[15] Gopalakrishnan V., Helmink B.A., Spencer C.N., Reuben A., Wargo J.A. (2018). The Influence of the Gut Microbiome on Cancer, Immunity, and Cancer Immunotherapy. Cancer Cell.

[16] Sjödahl G., Eriksson P., Liedberg F., Höglund M. (2017). Molecular classification of urothelial carcinoma: Global mRNA classification versus tumour-cell phenotype classification. J. Pathol..

[17] Andaluz Aguilar H., Iliuk A.B., Chen I., Tao W.A. (2020). Sequential phosphoproteomics and N-glycoproteomics of plasma-derived extracellular vesicles. Nat. Protoc..

[18] Tyson M.D., Barocas D.A. (2018). Quality of Life After Radical Cystectomy. Urol Clin North Am..

[19] Leue C., Kruimel J., Vrijens D., Masclee A., van Os J., Van Koeveringe G. (2016). Functional urological disorders: A sensitized defence response in the bladder–gut–brain axis. Nat. Rev. Urol..

[20] Worby C.J., Schreiber H.L., Straub T.J., van Dijk L.R., Bronson R.A., Olson B.S., Pinkner J.S., Obernuefemann C.L.P., Muñoz V.L., Paharik A.E. (2022). Longitudinal multi-omics analyses link gut microbiome dysbiosis with recurrent urinary tract infections in women. Nat. Microbiol..

[21] Badgeley A., Anwar H., Modi K., Murphy P., Lakshmikuttyamma A. (2020). Effect of probiotics and gut microbiota on anti-cancer drugs: Mechanistic perspectives. Biochim. et Biophys. Acta.

[22] Nowak A., Paliwoda A., Błasiak J. (2019). Anti-proliferative, pro-apoptotic and anti-oxidative activity of Lactobacillus and Bifidobacterium strains: A review of mechanisms and therapeutic perspectives. Crit. Rev. Food Sci. Nutr..

[23] He C., Li B., Huang L., Teng C., Bao Y., Ren M., Shan Y. (2020). Gut microbial composition changes in bladder cancer patients: A case-control study in Harbin, China. Asia Pacific J. Clin. Nutr..

[24] Larsen J.M. (2017). The immune response to Prevotella bacteria in chronic inflammatory disease. Immunology..

[25] Grivennikov S.I. (2012). Inflammation and colorectal cancer: Colitis-associated neoplasia. Semin. Immunopathol..

[26] Xu L., Wu L.-F., Deng F.-Y. (2019). Exosome: An Emerging Source of Biomarkers for Human Diseases. Curr. Mol. Med..

[27] Petrella G., Ciufolini G., Vago R., Cicero D.O. (2021). Urinary Metabolic Markers of Bladder Cancer: A Reflection of the Tumor or the Response of the Body?. Metabolites.

[28] Shi H., Li X., Zhang Q., Yang H., Zhang X. (2016). Discovery of urine biomarkers for bladder cancer via global metabolomics. Biomarkers.

[29] Ediriweera M.K., To N.B., Lim Y., Cho S.K. (2021). Odd-chain fatty acids as novel histone deacetylase 6 (HDAC6) inhibitors. Biochimie.

[30] Koal T., Klavins K., Seppi D., Kemmler G., Humpel C. (2015). Sphingomyelin SM(d18:1/18:0) is significantly enhanced in cerebrospinal fluid samples dichotomized by pathological amyloid-β42, tau, and phospho-tau-181 levels. J. Alzheimers Dis..

